# Supplementary material for: Nutritional Status Predicts Fatty Acid Uptake from Fish and Soybean Oil Supplements for Treatment of Cancer-Related Fatigue: Results from a Phase II Nationwide Study
Source: Nutrients. 2021 Dec 31;14(1):184. doi: 10.3390/nu14010184 (PMC8747418; doi:10.3390/nu14010184)
Supplement: Supplementary file 1 [file nutrients-14-00184-s001.zip › nutrients-1503220-supplementary.pdf]

Nutritional status predicts fatty acid uptake from fish and soybean oil supplements for treatment of cancer-related fatigue: Results from a phase II nationwide study

Kleckner et al.

Supplementary Table S1. *Associations between baseline nutritional status and change in fatigue as measured using the Multidimensional Fatigue Symptom Inventory-Short Form (MFSI; analysis of variance, n=80).*

	**Good nutritional status (n=55)**		**Light-moderate malnutrition (n=25)**		
**MFSI subscales**	**Mean**	**Std Dev**	**Mean**	**Std Dev**	**F-test p-value**
General	-3.33	5.41	-3.88	6.17	0.686
Physical	-2.84	3.86	-1.56	3.76	0.171
Emotional	-0.76	3.23	-1.96	3.69	0.146
Mental	-1.45	3.33	-1.24	2.49	0.774
Vigor	4.07	3.71	2.92	5.76	0.285
Total Fatigue	-12.46	12.93	-11.56	15.48	0.788
